# Shedding Light on Pediatric Fractures: Bridging the Knowledge Gap

**DOI:** 10.3390/children11050565

**Published:** 2024-05-08

**Authors:** Christiaan J. A. van Bergen, Joost W. Colaris

**Affiliations:** 1 Department of Orthopedic Surgery, Amphia Hospital, 4818 CK Breda, The Netherlands; 2 Department of Orthopaedics and Sports Medicine, Erasmus University Medical Center—Sophia Children’s Hospital, 3015 GD Rotterdam, The Netherlands; j.colaris@erasmusmc.nl

After the great success of the printed edition of the Special Issue “Pediatric Fractures—Volume I”, which was published in 2023 containing 24 high-quality papers [1], we are proud to present our new printed edition of the Special Issue “Pediatric Fractures—Volume II”.

Pediatric fractures, though commonplace, often find themselves in the shadows of adult orthopedic research. Despite their high prevalence [2,3,4], there remains a stark deficiency in comprehensive studies dedicated to understanding and effectively treating pediatric fractures. This is not merely a matter of academic interest, but carries significant implications for the health and well-being of children.

It is important to mention that children should not be treated as “miniature adults”. Unlike adults, children’s bones are still in the process of growth and development [5], making their fracture management markedly distinct. Children possess a remarkable ability to correct improperly healed fractures through growth—a phenomenon where nature itself acts as a friend in the healing process. However, there are instances where nature’s hand is less forgiving, particularly when fractures involve the growth plates. The physes are particularly vulnerable in children and must be carefully considered during treatment and follow-up [6]. Neglecting these factors can have far-reaching implications, potentially leading to growth disturbances and functional deficits in the affected limb [7,8,9].

Because of the unique potential of growth in pediatric bones, long-term follow-up is important to detect and address any potential complications that may arise over time, especially nowadays, to study the effects of the more aggressive treatment of pediatric fractures without substantiated evidence [10].

To address the gaps in knowledge concerning pediatric fractures, the Special Issue “Pediatric Fractures—Volume II” features eleven high-quality, peer-reviewed articles focusing on the comprehensive care of a variety of pediatric fractures.

This Special Issue places particular emphasis on fractures around the elbow and the associated complications that may arise. Saris et al. (1) focused on lateral humeral condyle fractures, which occur often in children but have potential pitfalls in treatment. The article describes classifications, treatment strategies, and handles on how to prevent complications. In line with this, Lewallen et al. (2) discuss acute elbow dislocation and their associated fractures in children and deliberate on conservative versus surgical treatment.

Even more frequent are pediatric supracondylar humerus fractures, which are often treated with closed reduction methods and percutaneous K-wire fixation. However, the configuration of the pins used to stabilize the fracture remains a point of debate. Therefore, Kaya et al. (3) conducted a randomized trial to determine the most effective pin configuration while also considering the potential risks associated with radiation exposure. Moreover, supracondylar humerus fractures are frequently associated with traumatic nerve injury. To unravel the best diagnostics and treatment of traumatic nerve palsies in these fractures, Graff et al. (4) wrote a review article of the current literature.

If nature does not behave like a friend in pediatric fractures and growth disturbances arise, clinicians are challenged to assess the indications and value of different treatment options. Siemensma et al. (5) dove into the unique treatment options of growth disturbances of the upper limb in children, which consist of a wait-and-see policy or surgical techniques, including physeal bar resection, (hemi-)epiphysiodesis, and osteotomies to correct the deformity. The pearls and pitfalls of these treatment strategies are discussed to guide clinicians.

In contrast with malunited wrist fractures, which remodel relatively quickly due to their proximity to the physis, diaphyseal forearm fractures are notorious for their malalignment and subsequent impairment of forearm rotation. In such a pathology, a corrective osteotomy is often performed. Roth et al. (6) assessed the accuracy of three-dimensional corrections and studied the relation between the precision of anatomic correction and functional outcome.

Moving from the upper to the lower extremity, interesting contributions were received that studied a spectrum of injuries from hip to foot. Michalik et al. (7) unraveled the treatment of aneurysmal bone cysts in the femoral neck and its relation to a pathological fracture in children.

Chandanani et al. (8) performed a comprehensive meta-analysis on the treatment of children with tibial spine avulsion fractures. Based on the results of 38 studies, they found that arthroscopic-assisted reduction and internal fixation with a suture seems to be the preferred treatment.

Patients with congenital tibial pseudoarthrosis are treated in different ways. Chou et al. (9) described three previously treated patients with congenital pseudoarthrosis of the tibia using a contralateral vascularized fibular bone graft and stabilization with a distal tibial locking plate.

Abnormal fibrous or bony connections between the tarsal bones of the foot occur in children and are known as tarsal coalitions. Although these coalitions do not always become symptomatic, some may cause pain and changes in morphology and biomechanics. Anastasio et al. (10) reviewed the foot biomechanics seen in tarsal coalitions and concomitant fractures. Furthermore, diagnostic and treatment options are discussed.

Finally, two studies on head trauma in children were published. Although these are common injuries, there is relatively little literature available on the subject. Palavani et al. (11) conducted a systematic review to unveil the state of the evidence concerning acute neurosurgical intervention, hospitalizations after injury, and neuroimaging in isolated skull fractures in children. In line with this research, Juncar et al. (12) studied the main clinical characteristics of pediatric facial fractures (such as fracture location, fracture pattern, treatment, complications, and evolution).

In conclusion, this Special Issue comprises a wide array of studies, all of which are aimed at enhancing the care of pediatric fractures. The editors are optimistic that this compilation will contribute significantly to closing knowledge gaps surrounding pediatric fractures. Furthermore, this Issue provides inspiration for further study and improved care for this challenging population.
